# Association of first-line thrombectomy technique and outcome in late-window large vessel occlusion strokes: A post hoc analysis of the MR CLEAN-LATE trial

**DOI:** 10.1177/17474930241268303

**Published:** 2024-07-31

**Authors:** Robrecht RMM Knapen, Susanne GH Olthuis, Adriaan CGM van Es, Bart J Emmer, Wouter J Schonewille, Christiaan van der Leij, Wim H van Zwam, Robert J van Oostenbrugge

**Affiliations:** 1 Department of Radiology and Nuclear Medicine, Maastricht University Medical Center+ and School for Cardiovascular Diseases (CARIM), Maastricht University, Maastricht, The Netherlands; 2 Department of Neurology, Maastricht University Medical Center+ and School for Cardiovascular Diseases (CARIM), Maastricht University, Maastricht, The Netherlands; 3 Department of Radiology, Leiden University Medical Center, Leiden, The Netherlands; 4 Department of Radiology and Nuclear Medicine, Amsterdam University Medical Center, Amsterdam, The Netherlands; 5 Department of Neurology, St. Antonius Nieuwegein, Nieuwegein, The Netherlands; 6 Department of Radiology and Nuclear Medicine, Maastricht University Medical Center+, Maastricht, The Netherlands

**Keywords:** Stroke, intra-arterial thrombectomy, first-line technique, late-window

## Abstract

**Background:**

This study aimed to compare direct aspiration, stent retriever, and the combined thrombectomy technique on clinical, safety, and technical outcomes in late-window stroke patients included in the MR CLEAN-LATE trial.

**Methods:**

This post hoc analysis of the MR CLEAN-LATE trial included patients treated with direct aspiration, stent retriever, or combined thrombectomy technique as first-line approach. Primary outcome was the modified Rankin Scale (mRS) score at 90 days follow-up, and compared between the three groups with ordinal logistic regression analysis. Secondary outcomes included mortality at 90 days, total technique switches, procedure time, recanalization rate measured with the expanded thrombolysis in cerebral infarction (eTICI) score, and symptomatic intracranial hemorrhage (sICH). Predefined variables were used for adjustments.

**Results:**

In the MR CLEAN-LATE trial, 258 patients underwent endovascular treatment and 232 were included in our analyses. The mRS at 90 days did not differ (stent retriever vs. direct aspiration: adjusted common odds ratio (acOR) = 1.35, 95% confidence interval (CI) = 0.73 to 2.50; stent retriever vs. combined: acOR = 1.13, 95% CI = 0.64 to 2.00; direct aspiration vs. combined: acOR = 1.19, 95% CI = 0.64 to 2.21). Direct aspiration thrombectomy was accompanied with more switches to another technique compared to the stent retriever (adjusted odds ratio (aOR) = 6.50, 95% CI = 2.52 to 16.8) or combined group (aOR = 4.67, 95% CI = 1.80 to 12.1) and with higher sICH rates compared to the combined technique (13% vs. 2.5%; aOR = 8.19, 95% CI = 1.49 to 45.1). Mortality, procedure time, and eTICI did not differ.

**Conclusion:**

Stent retriever, direct aspiration, or the combined thrombectomy technique as first-line approach showed no differences in clinical outcome in late-window stroke patients. Direct aspiration was accompanied with higher sICH rates and more switcher to another technique compared to the combined group.

## Introduction

Three commonly used endovascular thrombectomy (EVT) techniques for treatment of patients with ischemic stroke due to large vessel occlusion (LVO) are: direct aspiration, stent retriever thrombectomy, and the combination of both. Equal clinical outcomes are reported between direct aspiration and stent retriever thrombectomy as first-line technique within the early window (patients treated within 6 h of stroke onset).^1–4^ The DAWN and DEFUSE-3 trials showed that patients, selected on clinical and perfusion criteria, benefit from EVT between 6 and 24 h after stroke onset (late window) as well.^5,6^ Recently, the MR CLEAN-LATE showed that a significant treatment benefit was present if late-window patients were selected on the presence of collateral flow on computed tomography-angiography (CTA).

Literature on the preferred thrombectomy technique in these late-window patients is limited. In addition, it is known that older thrombi have a different composition than younger, fresher thrombi. Older thrombi contain fewer erythrocytes compared to fresh thrombi.^7^ A small experimental study suggested differences in thrombectomy efficacy between erythrocyte-rich and fibrin-rich clots. Direct aspiration was associated with fewer passes when retrieving erythrocyte-rich clots, whereas stent retriever thrombectomy resulted in fewer distal thrombi in fibrin-rich clots.^8^ This difference may lead to distinct outcomes between the three thrombectomy techniques in late-window stroke patients.

## Aim

Therefore, the aim of this study is to investigate the clinical, technical, and safety outcomes between direct aspiration, stent retriever thrombectomy, and the combined thrombectomy technique as first-line technique in late-window patients, who were included in the MR CLEAN-LATE trial.

## Methods

### Design and participants

This post hoc analysis included patients treated with EVT in the MR CLEAN-LATE, between February 2018 and January 2022.^9^ The MR CLEAN-LATE trial was a multicenter, open-label, randomized, controlled trial, which compared EVT with best medical care in patients with ischemic stroke due to LVO in the anterior circulation who presented between 6 and 24 h after stroke onset or last seen well in patients selected based on the presence of collateral flow. Ethical approval has been approved by the medical ethics committee of Erasmus MC University Medical Center (MEC-2017-367). The MR CLEAN-LATE protocol and inclusion and exclusion criteria have been described previously.^10^ Patients included in this study had at least one thrombectomy attempt and a proven anterior circulation stroke (internal carotid artery (ICA), ICA-terminus (ICA-T), or middle cerebral artery (M1, M2)) on CTA. Patients were excluded in case details on first-line thrombectomy techniques were missing or when they had an anterior cerebral artery occlusion.

### Treatment

Patients were treated after 6 h and within 24 h after stroke onset or last seen well. Treating physicians were free to choose the devices and first-line thrombectomy technique during EVT. When a distal access catheter (DAC) was used, we assumed aspiration, unless the DAC was unknown or no aspiration was mentioned specifically. When a treating physician used another technique after the first attempt, it was registered as a thrombectomy technique switch. Patients were divided into three groups based on the first-line thrombectomy technique: stent retriever thrombectomy, direct aspiration thrombectomy, or combined technique.

### Outcome measures

The primary outcome was the score on the entire distribution of the modified Rankin Scale (mRS) at 90 days follow-up. The mRS ranges from 0 (no disability) to 6 (death).^11^ Secondary outcomes included excellent and favorable functional outcomes (mRS 0–1 vs. 2–6 and mRS 0–2 vs. 3–6, respectively), mortality at 90 days follow-up, the National Institutes of Health Stroke Score (NIHSS) at 24 h after EVT, early neurological improvement, reperfusion grade after EVT, procedure time, procedural complications, total attempts, total technique switches, successful recanalization with one or more attempts without switching to another thrombectomy technique, and symptomatic intracranial hemorrhages (sICHs) within 72 h after EVT.

The NIHSS was scored at 24 h after EVT. The NIHSS represents stroke severity and ranges from 0 (no deficit) to 42 (maximum deficit). Early neurological improvement was defined as improvement of 4 or more points on the NIHSS at 24 h. The expanded thrombolysis in cerebral infarction (eTICI) score was used to define the reperfusion grade. A scale ranges from 0 (no reperfusion) to 3 (complete reperfusion).^12^ eTICI ⩾2B and eTICI ⩾2C were defined as successful and excellent reperfusion grade, respectively. The procedure time was the time between groin puncture and recanalization or last contrast injection. An sICH was defined as an intracranial hemorrhage and when the patient had neurological deterioration (two points increase in one subcategory on the NIHSS or an increase of four or more points in total in the NIHSS score), which was related to the hemorrhage (according to the Heidelberg Bleeding Classification).^13^

### Imaging assessment

All images at baseline and follow-up were assessed and scored by a core laboratory, which were blinded for clinical information. The collateral status (range from no collaterals (0) to good collaterals (3)) and the Acute Stroke Prognosis Early CT Score (ASPECTS, a 10-point scale in which every region not affected by ischemia reflects 1 point) were both scored on baseline CTA and CT, respectively.^14,15^

### Statistical analysis

Baseline characteristics and outcomes were presented with standard statistics. The Pearson’s chi-square test or Fisher’s exact test were used to compare ordinal and dichotomous data; for continuous data, we used the analysis of variance (ANOVA) test or Kruskal–Wallis test after checking histograms on distribution. For the mRS score, we used a multiple ordinal logistic regression analysis to compare the effect of the first-line thrombectomy technique. We compared the direct aspiration group and combined technique group to the stent retriever thrombectomy group (comparator), and the direct aspiration group to the combined technique group (comparator). Results were presented using a common odds ratio (cOR) with a corresponding 95% confidence interval (CI). Binary and linear outcomes were tested using multiple linear or binary logistic regression analyses as appropriate. Continuous outcomes were checked for normality of distribution of the residuals using Q-Q plots. When normality was observed, the outcome was transformed using the natural logarithm. Relative percentages were afterwards calculated using the following formula: (exponentiate (coefficient))*100%. Three sensitivity analyses were performed. The first sensitivity analysis was, after removing patients with an M2-occlusion, to evaluate the effect of the three different first-line thrombectomy techniques on the mRS score at 90 days. The second sensitivity analysis was, after selecting patients with successful reperfusion within one or more attempts without switching to another technique, to evaluate the thrombectomy techniques on the sICH and total attempt rates. The third sensitivity analysis was an inverse probability of treatment weighting (IPTW) with propensity score method analysis to balance baseline patient characteristics. The same adjustments were used as for our primary analysis.

All regression analyses were adjusted for: age, baseline NIHSS, baseline mRS (0 vs. 1–5), time between onset symptoms and groin puncture, ASPECTS score, and (un)witnessed stroke. The analyses were performed using R (version 4.3.1). The alpha was set at 5%.

### Missing values

All descriptive statistics were presented using raw data. Missing data in the outcome measures were imputed for regression analyses with multiple imputation with chained equations (MICEs) using the *mice* package (version 3.16.0). Predefined variables were used, and the number of imputations was 50. The missing data are presented in Table 1 for the baseline variables and in Table 2 for the outcome measurements. The mRS was not missing in any patient, and the NIHSS at 24 h was in 2.5% (n = 5) of the patients.

**Table 1.** Baseline characteristics between the three different first-line thrombectomy approaches.

	SR only (n = 87)		ASP only (n = 64)		SR + ASP (n = 81)		p	Missing (%)
Age—mean (SD)	68.7	(13.1)	71.8	(12.6)	72.8	(11.9)	0.09	0
Male sex—n (%)	34	(39)	29	(45)	38	(47)	0.56	0
NIHSS—median [IQR]	10	[6.0–18]	14	[7.0–19]	7.0	[5.0–14]	**<0.01**	3.9
IVT given—n (%)	2	(2.3)	4	(6.3)	3	(3.7)	0.48	0
Systolic blood pressure—mean mm Hg (SD)	156	(29)	155	(27)	154	(29)	0.89	0
Medical history—n (%)								
Pre-mRS—n (%)							0.15	0
0	59	(68)	38	(59)	39	(48)		
1	18	(21)	19	(30)	25	(31)		
2	9	(10)	7	(11)	16	(20)		
>2	1	(1.1)	0	(0)	1	(1.2)		
Ischemic stroke	15	(17)	13	(20)	18	(22)	0.74	0.4
Atrial fibrillation	15	(17)	11	(17)	19	(24)	0.53	0.4
Hypertension	43	(50)	40	(63)	48	(59)	0.26	0.4
Diabetes mellitus	10	(12)	7	(11)	13	(16)	0.59	0.4
History of smoking	24	(29)	18	(32)	14	(18)	0.12	5.6
Usage of coumarin	7	(8.0)	9	(14)	11	(14)	0.42	0
Usage of anticoagulation	5	(5.7)	2	(3.1)	9	(11)	0.17	0
Usage of antiplatelet	26	(30)	15	(23)	27	(33)	0.43	0
Imaging								
Collaterals—n (%)							0.65	1.7
Grade 0	6	(7.1)	3	(4.8)	2	(2.5)		
Grade 1	18	(21)	16	(25)	24	(30)		
Grade 2	37	(44)	31	(49)	35	(44)		
Grade 3	24	(28)	13	(21)	19	(24)		
ASPECTS—median [IQR]	9.0	[8.0–10]	8.0	[6.8–10]	9.0	[7.0–10]	0.49	0
Occlusion location on CTA—n (%)							0.52	0
ICA	1	(1.1)	2	(3.1)	2	(2.5)		
ICA-T	11	(13)	11	(17)	8	(9.9)		
MCA segment M1	44	(51)	37	(58)	45	(56)		
MCA segment M2	31	(36)	14	(22)	26	(32)		
Tandem lesion—n (%)	13	(16)	17	(27)	17	(21)	0.26	2.6
Workflow								
Transfer from primary stroke center—n (%)	47	(54)	37	(58)	50	(62)	0.60	
Onset to groin—median (min) [IQR]	719	[555–935]	696	[563–860]	810	[590–997]	0.06	1.3
Balloon guide catheter used—n (%)	69	(95)	22	(37)	62	(79)	**<0.001**	9.1

SR: stent retriever thrombectomy; ASP: direct aspiration thrombectomy; NIHSS: National Institutes of Health Stroke Scale; IVT: intravenous thrombolysis; mRS, modified Rankin Scale; ASPECTS: Alberta Stroke Program Early CT Score; CTA: CT-angiography; ICA: internal carotid artery; ICA-T: internal carotid artery terminus; MCA: middle cerebral artery; M1: horizontal segment of the middle cerebral artery; M2: insular segment of the middle cerebral artery.

**Table 2.** Outcome measures between the three groups.

	SR only (n = 87)		ASP only (n = 64)		SR + ASP (n = 81)	
mRS at 90 days—n (%)						
0	9	(10)	8	(13)	3	(3.7)
1	11	(13)	8	(13)	10	(12)
2	15	(17)	9	(14)	22	(27)
3	10	(12)	6	(9.4)	12	(15)
4	11	(13)	9	(14)	9	(11)
5	14	(16)	8	(13)	8	(9.9)
6	17	(20)	16	(25)	17	(21)
mRS score 0–1—n (%)	20	(23)	16	(25)	13	(16)
mRS score 0–2—n (%)	35	(40)	25	(39)	35	(43)
Successful reperfusion (eTICI ⩾2B)—n (%)	70/85	(82)	50/62	(81)	56/81	(69)
Excellent reperfusion (eTICI ⩾2 C)—n (%)	49/85	(58)	38/62	(61)	40/81	(49)
Complete reperfusion (eTICI = 3)—n (%)	33/85	(39)	18/62	(29)	28/81	(35)
Mortality at 90 days—n (%)	17	(20)	16	(25)	17	(21)
NIHSS 24 h post-EVT^a^—median [IQR]	6.0	[2.5–13]	8.0	[3.0–18]	5.0	[3.0–15]
Early improvement—n (%)	36/81	(44)	26/62	(42)	27/75	(36)
Procedure time^b^—median (min) [IQR]	52	[34–70]	55	[34–74]	51	[33–80]
First-attempt successful (eTICI = 3)—n (%)	21/84	(25)	9/62	(15)	20/81	(25)
Per procedural complications—n (%)	6/86	(7.0)	3/64	(4.7)	3/81	(3.7)
sICH—n (%)	7	(8.0)	8	(13)	2	(2.5)
Total attempts^c^—median [IQR]	2	[1–3]	2	[1–3]	1	[1–3]
Switching thrombectomy techniques—n (%)	8	(9.2)	25	(39)	8	(10)
Successful reperfusion without switching—n (%)	63/85	(74)	32/62	(52)	52	(64)

SR: stent retriever thrombectomy; ASP: direct aspiration thrombectomy; mRS, modified Rankin Scale; eTICI, expanded treatment in cerebral ischemia; NIHSS: National Institutes of Health Stroke Scale.

a NIHSS was missing in five patients.

b Procedure time was missing in seven patients.

c Total attempts were missing in one patient.

## Results

### Baseline characteristics

**Figure 1.**
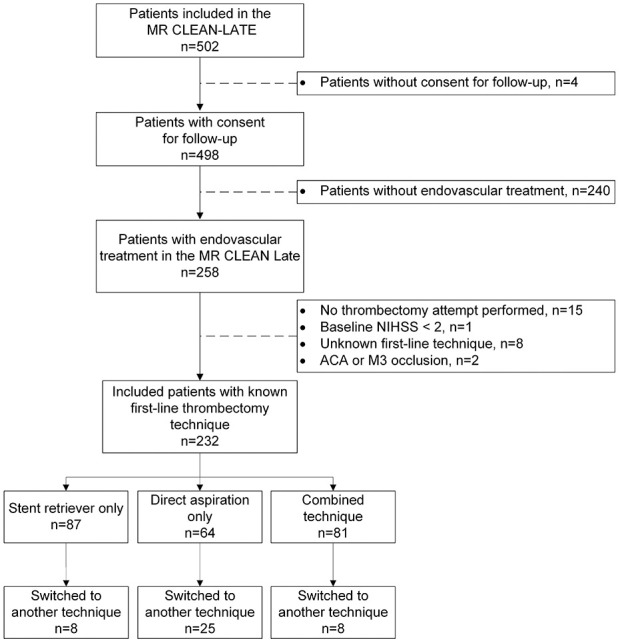
Flowchart of included patients.

In the MR CLEAN-LATE trial, 502 patients were included. After applying the inclusion and exclusion criteria, a total of 232 patients were included in this post hoc analysis (Figure 1). Eighty-seven (38%) of these patients were treated with stent retriever thrombectomy, 64 (28%) with direct aspiration thrombectomy, and 81 (35%) with the combined technique. Baseline patient characteristics are shown in Table 1. The NIHSS score at baseline was different between the groups; patients treated with the combined thrombectomy technique had the lowest NIHSS (median = 7, 95% CI = (5.0 to 14)).

#### Clinical outcome

**Figure 2.**
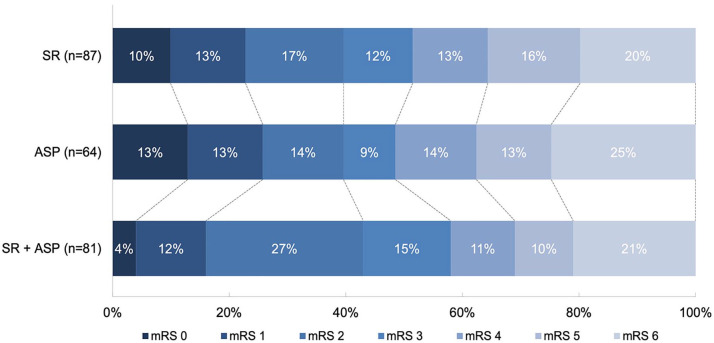
The distribution of the modified Rankin Scale at 90 days follow-up between patients treated with stent retriever thrombectomy, direct aspiration thrombectomy, or combined technique. Multivariable logistic regression with adjustments showed no differences between stent retriever and direct aspiration thrombectomy (adjusted common odd ratio (acOR) = 1.35, 95% CI = 0.73 to 2.50) and combined technique (acOR = 1.13, 95% CI = 0.64 to 2.00) and between direct aspiration thrombectomy and combined technique (acOR = 1.19, 95% CI = 0.64 to 2.21).

No differences were observed in mRS score at 90 days in patients treated with direct aspiration thrombectomy (adjusted common odds ratio (acOR) = 1.35, 95% CI = 0.73 to 2.50) or combined technique (acOR = 1.13, 95% CI = 0.64 to 2.00) compared to the stent retriever thrombectomy group or aspiration thrombectomy compared to combined technique (acOR = 1.19, 95% CI = 0.64 to 2.21) (Figure 2). Excellent and favorable functional outcomes, the NIHSS score at 24 h, and the mortality at 90 days also did not differ between the groups (Tables 2 and 3).

**Table 3.** Associations between clinical, safety, and technical outcomes between the three first-line thrombectomy approaches.

	EE	ASP vs. SR	SR + ASP vs. SR	ASP vs. SR + ASP
mRS at 90 days*	acOR	1.35 (0.73 to 2.50)	1.13 (0.64 to 2.00)	1.19 (0.64 to 2.21)
mRS 0–1 at 90 days	aOR	1.27 (0.55 to 2.92)	0.57 (0.24 to 1.34)	2.24 (0.90 to 5.57)
mRS 0–2 at 90 days	aOR	1.32 (0.59 to 2.95)	1.62 (0.75 to 3.54)	0.81 (0.35 to 1.87)
Post eTICI score	acOR	0.86 (0.47 to 1.55)	0.77 (0.42 to 1.40)	1.11 (0.60 to 2.07)
Successful reperfusion (eTICI ⩾2B)	aOR	0.76 (0.32 to 1.83)	0.50 (0.23 to 1.10)	1.53 (0.67 to 3.51)
Excellent reperfusion (eTICI ⩾2C)	aOR	1.15 (0.57 to 2.34)	0.87 (0.44 to 1.71)	1.32 (0.64 to 2.72)
Complete reperfusion (eTICI = 3)	aOR	0.67 (0.32 to 1.40)	0.97 (0.48 to 1.94)	0.69 (0.32 to 1.49)
Mortality at 90 days	aOR	0.84 (0.31 to 2.23)	0.76 (0.29 to 2.00)	1.10 (0.39 to 3.05)
NIHSS 24 h post-EVT	aß	1.03 (−1.38 to 3.44)	0.27 (−2.10 to 2.63)	0.76 (−1.79 to 3.31)
Early improvement	aOR	0.87 (0.43 to 1.79)	1.04 (0.52 to 2.10)	0.84 (0.39 to 1.78)
Procedure time	a%	1.6 (−16 to 23)	3.9 (−13 to 25)	−2.3 (−20 to 19)
First-attempt successful (eTICI = 3)	aOR	0.82 (0.40 to 1.68)	0.87 (0.43 to 1.73)	0.95 (0.45 to 2.01)
Per procedural complications	aOR	0.80 (0.17 to 3.69)	0.59 (0.13 to 2.79)	1.36 (0.24 to 7.51)
sICH	aOR	1.63 (0.51 to 5.20)	0.20 (0.04 to 1.09)	8.19 (1.49 to 45.1)^#^
Total attempts	aß	0.48 (−0.03 to 1.00)	0.03 (−0.47 to 0.52)	0.46 (−0.08 to 0.99)
Switching techniques	aOR	6.50 (2.52 to 16.8)^$^	1.39 (0.46 to 4.20)	4.67 (1.80 to 12.1)^^^
Successful reperfusion without switching	aOR	0.35 (0.17 to 0.72)^^^	0.57 (0.28 to 1.16)	0.61 (0.30 to 1.25)

ASP: direct aspiration thrombectomy; SR: stent retriever thrombectomy; mRS: modified Rankin Scale; eTICI: expanded treatment in cerebral ischemia; NIHSS: National Institutes of Health Stroke Scale; acOR: adjusted common odds ratio; aOR: adjusted odds ratio; EE: effect estimate; a%: adjusted percentage; aß: adjusted beta-coefficient.

* Common odds ratio for improved mRS score: ^#^p < 0.05; ^^^p < 0.01; ^$^p < 0.001.

#### Technical outcome

Treating physicians switched more often to another thrombectomy technique when they started with direct aspiration thrombectomy compared to stent retriever thrombectomy (47% vs. 22%; adjusted odds ratio (aOR) = 6.50, 95% CI = 2.52 to 16.8) or to the combined technique (47% vs. 20%; aOR = 4.67, 95% CI = 1.80 to 12.1). Supplemental Table S1 provides an overview of the second thrombectomy approaches. Successful reperfusion rate was the lowest in the combined group (69%) compared to the stent retriever group and direct aspiration group (82% and 81%), although not statistically significant. Successful reperfusion without switching to another technique was lower in the direct aspiration group compared to the stent retriever group (52% vs. 74%; aOR = 0.35, 95% CI = 0.17 to 0.72). Excellent, complete, and first-attempt successful recanalization rates were not significantly different (Tables 2 and 3).

#### Safety outcomes

Although the per-procedural complication rates were lower in the direct aspiration group (4.7%) and the combined technique group (3.7%) compared to the stent retriever group (7.0%), it did not statistically differ (aOR = 0.80, 95% CI = 0.17 to 3.69 and aOR = 0.59, 95% CI = 0.13 to 2.79, respectively). Patients treated with direct aspiration thrombectomy had higher sICH rates compared to the combined technique (13% vs. 2.5%; aOR = 8.19, 95% CI = 1.49 to 45.1).

#### Sensitivity analysis

In total, 71 patients had an M2-occlusion. Sensitivity analysis excluding the M2 occlusions showed no differences in mRS scores at 90 days between the three different first-line thrombectomy techniques (Supplemental Table S2). When the patients with successful reperfusion without switching to another thrombectomy technique were compared, no differences were seen in both sICH rates and total attempts (Supplemental Table S3).

IPTW was performed to balance the three groups on baseline NIHSS and balloon guide catheter (BGC) use during thrombectomy. Results regarding sICH rates between direct aspiration and the combined technique, as well as the switch rates to other techniques, were similar in the IPTW analysis compared to the main analysis (Supplemental Table S4). The IPTW analysis showed higher rates of total attempts in the direct aspiration group compared to the stent retriever group (aß = 0.69, 95% CI = 0.13 to 1.25) and the combined group (aß = 0.66, 95% CI = 0.10 to 1.22).

## Discussion

This MR CLEAN-LATE post hoc analysis compared the functional, technical, and safety outcomes for three different thrombectomy techniques. No significant differences were observed among these groups in terms of clinical outcome. sICH rates were higher in the direct aspiration group compared to the combination group; however, numbers were very small. Treating physicians switched significantly more often to another thrombectomy technique when direct aspiration thrombectomy was the first-line thrombectomy technique.

After adjustment for the baseline NIHSS and after balancing in the IPTW analysis, the clinical outcomes did not differ between groups. The average favorable functional outcome was 41%, aligning with studies performing EVT in late-window patients, with mRS 0–2 scores ranging between 30% and 48%.^16–22^ In the DAWN and DEFUSE-3 late-window trials, this percentage was slightly higher with 45–59%. However, patients were selected more strictly in these trials.^6,23^ None of the abovementioned studies conducted direct comparisons of different first-line thrombectomy techniques. Nevertheless, meta-analyses and trials presented comparable clinical outcomes among the thrombectomy techniques in early-window patients.^24^ In addition, studies comparing early versus late-window patients concluded similar clinical outcomes between both timeframes.^16,18,21^ These results substantiate the theory that overall there are no differences in clinical outcome between treating strategies in both early and late-window stroke patients.

In our study, stent retriever was relatively more used in patients presenting with an M2-occlusion: 44% compared to 20% direct aspiration and 37% combined. It is acknowledged that late-window patients with an M2-occlusion generally have better clinical outcomes compared to M1-occlusions.^25^ This might overestimate the results of the stent retriever group in our study. Therefore, we performed a sensitivity analysis without patients with a M2-occlusion, revealing no differences in mRS scores between the three thrombectomy technique groups (Supplemental Table S2). This makes our conclusion more robust. The higher prevalence of M2 occlusions in the stent retriever group may be the reason why this technique is the most used in the MR CLEAN-LATE. Some treating physicians might assume an increased popularity of direct aspiration or the combined technique over the years and a decrease of stent retriever thrombectomy only. Supplemental Figure S1 overviews the use of the techniques over the years, showing a small decrease in stent retriever use and increase of combined technique use.

Direct aspiration thrombectomy was the most common technique to be switched from after the first attempt (61%), particularly to the combined thrombectomy technique (84%) (Supplemental Table S1). Direct aspiration may be considered as the least invasive thrombectomy technique; therefore, switching from aspiration to the combined group may be considered as a common practice pattern. Another potential reason for this may be the presence of thrombi more distally after direct aspiration only or because of pragmatic reasons, since the aspiration catheter is already in use. However, numbers are too small to draw solid conclusions.

Although the procedural complications were comparable between the three techniques, the incidence of sICH was the highest in the direct aspiration group. This result is not in line with most literature, since studies present comparable sICH rates in patients treated with stent retriever thrombectomy and direct aspiration thrombectomy.^26–28^ One other study reported lower sICH rates in patients treated with combined thrombectomy technique compared to direct aspiration thrombectomy.^29^ Since the CIs are wide, solid conclusions cannot be drawn. However, the sICH rates may influence the clinical outcomes when larger groups are compared. In addition, the IPTW analysis revealed higher sICH rates in the direct aspiration group, as well as more attempts and more switches to another techniques compared to the other two techniques. The combination of more attempts and more often switches to other techniques might explain the higher sICH rates. It is known that more attempts results in higher changes of sICHs.^30^ This theory can be substantiated by the fact that the sensitivity analysis after selecting patients with successful recanalization without switching to another thrombectomy technique did not show any differences in sICH rates and in total attempts between the three groups.

Certain limitations need to be mentioned. First, the thrombectomy technique approach was at the discretion of the treating physician, potentially introducing bias. Second, a variety of different aspiration catheters and stent retrievers were used during the years of the MR CLEAN-LATE, and the differences between these devices are not thoroughly investigated. Third, certain thrombus characteristics, such as thrombus length and density on imaging, are not taken into regard. These characteristics may influence the technical success of thrombectomy techniques; however, the influence on clinical outcome seems to be limited.^31,32^ On the contrary, strengths of the study are first that data were collected prospectively as part of the MR CLEAN-LATE trial. Second, we differentiated between stent retriever thrombectomy only and combined thrombectomy technique, whereas most literature defined both groups as stent retriever thrombectomy. Third, our data represent real-world daily practice with a limited number of missing data and no missings in the primary outcome, contributing to the generalizability of our analyses to other stroke centers in late-window stroke patients.

As in the early window, stent retriever thrombectomy, direct aspiration thrombectomy, or the combined technique as first-line technique showed no significant differences in clinical outcome in late-window stroke patients. Direct aspiration was accompanied with higher sICH rates and higher switch rates to another technique compared to the combined group.

## Supplemental Material

sj-docx-1-wso-10.1177_17474930241268303 – Supplemental material for Association of first-line thrombectomy technique and outcome in late-window large vessel occlusion strokes: A post hoc analysis of the MR CLEAN-LATE trial Supplemental material, sj-docx-1-wso-10.1177_17474930241268303 for Association of first-line thrombectomy technique and outcome in late-window large vessel occlusion strokes: A post hoc analysis of the MR CLEAN-LATE trial by Robrecht RMM Knapen, Susanne GH Olthuis, Adriaan CGM van Es, Bart J Emmer, Wouter J Schonewille, Christiaan van der Leij, Wim H van Zwam and Robert J van Oostenbrugge in International Journal of Stroke
