# Supplementary material for: Association of first-line thrombectomy technique and outcome in late-window large vessel occlusion strokes: A post hoc analysis of the MR CLEAN-LATE trial
Source: Int J Stroke. 2024 Jul 31;19(10):1113–22. doi: 10.1177/17474930241268303 (PMC11590395; doi:10.1177/17474930241268303)
Supplement: sj-docx-1-wso-10.1177_17474930241268303 – Supplemental material for Association of first-line thrombectomy technique and outcome in late-window large vessel occlusion strokes: A post hoc analysis of the MR CLEAN-LATE trial [file sj-docx-1-wso-10.1177_17474930241268303.docx]

**Supplemental material**

**Figure S1.** Overview of used first-line thrombectomy technique over the years.

ASP, direct aspiration thrombectomy; SR, stent retriever thrombectomy.

* The years are based on the inclusion time of the MR CLEAN LATE trial. Year 1 February 2018-January 2019; Year 2 February 2019-January 2020; Year 3 February 2020-January 2021; Year 4 February 2021-January 2022.

**Table S1.** Second technique approaches after first-line technique switch

|  | SR  (n=8) | | ASP  (n=25) | | SR+ASP  (n=8) | |
| --- | --- | --- | --- | --- | --- | --- |
| Second technique approach – n. (%) | | | | | | |
| SR | NA | NA | 4 | (16) | 3 | (38) |
| ASP | 3 | (38) | NA | NA | 5 | (63) |
| SR+ASP | 5 | (63) | 21 | (84) | NA | NA |

ASP, direct aspiration thrombectomy; SR, stent retriever thrombectomy.

**Table S2.** Sensitivity analysis without M2-occlusions

|  | EE | ASP versus SR | SR+ASP versus SR | ASP versus SR+ASP |
| --- | --- | --- | --- | --- |
| mRS at 90 days* | acOR | 1.64 (0.77-3.48) | 1.21 (0.58-2.53) | 1.34 (0.64-2.81) |
| mRS 0-1 at 90 days | aOR | 1.47 (0.53-4.14) | 0.53 (0.16-1.74) | 2.77 (0.86-8.94) |
| mRS 0-2 at 90 days | aOR | 1.59 (0.59-4.28) | 1.75 (0.64-4.76) | 0.91 (0.33-2.51) |

ASP, direct aspiration thrombectomy; SR, stent retriever thrombectomy; mRS, modified Rankin Scale; eTICI.

**Table S3.** Sensitivity analysis in patients with successful recanalization with one or more attempts without switching to another technique

|  | EE | ASP versus SR | SR+ASP versus SR | ASP versus SR+ASP |
| --- | --- | --- | --- | --- |
| sICH | aOR | 1.10 (0.25-4.78) | 2.26 (0.03-1.34) | 4.84 (0.70-33.6) |
| Total attempts | aß | 0.14 (-0.42-0.70) | 0.16 (-0.35-0.67) | -0.02 (-0.62-0.58) |

ASP, direct aspiration thrombectomy; SR, stent retriever thrombectomy; mRS, modified Rankin Scale; eTICI. ^*^p<0.05; ^#^p<0.01; ^^^p<0.001

**Table S4.** Inverse probability of treatment weighting analysis

|  | EE | ASP versus SR | SR+ASP versus SR | ASP versus SR+ASP |
| --- | --- | --- | --- | --- |
| mRS at 90 days* | acOR | 0.90 (0.64-1.28) | 0.98 (0.70-1.38) | 0.92 (0.66-1.29) |
| mRS 0-1 at 90 days | aOR | 0.96 (0.40-2.35) | 0.44 (0.15-1.27) | 2.17 (0.78-6.07) |
| mRS 0-2 at 90 days | aOR | 0.84 (0.39-1.80) | 1.61 (0.73-3.54) | 0.52 (0.23-1.16) |
| Post eTICI score | acOR | 0.84 (0.60-1.19) | 0.81 (0.55-1.18) | 1.05 (0.74-1.48) |
| Successful reperfusion (eTICI ≥2B) | aOR | 1.30 (0.52-3.23) | 0.59 (0.26-1.35) | 2.21 (0.95-5.11) |
| Excellent reperfusion (eTICI ≥2C) | aOR | 0.97 (0.48-1.96) | 0.67 (0.33-1.37) | 1.44 (0.72-2.90) |
| Complete reperfusion  (eTICI = 3) | aOR | 0.52 (0.24-1.16) | 1.14 (0.53-2.46) | 0.46 (0.21-1.02) |
| Mortality at 90 days | aOR | 1.32 (0.48-3.67) | 1.09 (0.40-2.97) | 1.22 (0.47-3.17) |
| NIHSS 24h post-EVT | aß | 1.63 (-0.70 – 3.95) | 0.20 (-2.27 – 2.68) | 1.42 (-0.98-3.82) |
| Early improvement | aOR | 0.80 (0.38-1.65) | 0.96 (0.45-2.05) | 0.83 (0.39-1.75) |
| Procedure time | a% | 0.4 (-17 – 21) | 6.2 (-12 – 28) | -5.4 (-22 – 14) |
| First-attempt successful (eTICI = 3) | aOR | 0.63 (0.31-1.30) | 0.77 (0.37-1.59) | 0.83 (0.40-1.72) |
| Per procedural complications | aOR | 1.08 (0.22-5.33) | 0.92 (0.18-4.71) | 1.17 (0.23-5.93) |
| sICH | aOR | 1.87 (0.60-5.81) | 0.32 (0.07-1.34) | **5.91 (1.44-24.3)^^^** |
| Total attempts | aß | **0.69 (0.13 – 1.25)^#^** | 0.03 (-0.55-0.61) | **0.66 (0.10-1.22)^#^** |
| Switching techniques | aOR | **9.45 (3.54-25.2)^$^** | 1.44 (0.46-4.53) | **6.56 (2.52-17.1)^$^** |
| Successful recanalization without switching | aOR | **0.35 (0.17-0.73)^^^** | 0.62 (0.29-1.33) | 0.56 (0.28-1.13) |

*Common odds ratio for improved mRS score; ^#^p<0.05; ^^^p<0.01; ^$^p<0.001
